# A study on the effective management of pain in major traumas

**DOI:** 10.1186/1757-7241-22-S1-P14

**Published:** 2014-07-07

**Authors:** Kevin Dodd, Anthony Hudson, Heather Jarman

**Affiliations:** 1

## Background

Pain associated with major trauma often remains uncontrolled by medical professionals in Emergency Departments (ED). This study was established in an attempt to gauge the efficacy of pain control in major traumas by determining the time taken to get patients pain under control.

The study also aimed to look at the types of analgesia being used and the efficacy of pre-hospital analgesia.

## Methods

Only patients that triggered the activation of the Major Trauma team were included in the study.

Over a 3 week period, patients meeting the inclusion criteria were questioned on arrival and initial pain score was taken. Information about the presenting complaint and en-route medication was also recorded. Patients were followed up every 10 minutes with their pain score, analgesia, procedures and treatments recorded. The patients were followed up until such a time as they reported two consecutive pain scores of 2 or until they reported that their pain was sufficiently controlled.

## Results

A total of 16 people were included in the study, 8 males and 8 females.

On average it took 80 minutes to achieve pain control with females reporting a higher time of 87.5 minutes compared to 74.5 minutes in males. The average time for pain control was 116 minutes for patients receiving IV morphine only, 61 minutes for patients receiving IV paracetamol only, 135 minutes for those receiving both IV paracetamol and IV morphine and 85 minutes for those receiving oral analgesia only.

The average pain score on arrival to the ED was 6.06.

## Conclusions

This study highlights the need for faster and better intervention for pain control in Major Traumas.

Several methods could be implemented to achieve this:

•Implementing more regular pain scoring.

•The possible use of alternative pain medication or nerve blocks.

•Recommend a review of pain management in pre-hospital services.

